# Dynamic Motion and Rearranged Molecular Shape of Heme in Myoglobin: Structural and Functional Consequences

**DOI:** 10.3390/molecules18033168

**Published:** 2013-03-11

**Authors:** Saburo Neya

**Affiliations:** Department of Physical Chemistry, Graduate School of Pharmaceutical Sciences, Chiba University, Chuoh-Inohana, Chiba City, Chiba 260-8675, Japan; E-Mail: sneya@faculty.chiba-u.jp

**Keywords:** host-guest chemistry, globin pocket, dynamic heme rotation, heme deformation, O_2_ binding

## Abstract

Myoglobin, a simple oxygen binding protein, was reconstituted with various types of synthetic hemes to manipulate the heme-globin interactions. From the paramagnetic NMR analysis, small heme was found to rotate rapidly about the iron-histidine bond upon. This is a novel and typical example for the fluctuation of protein. The dynamic NMR analysis indicated that the 360° rotational rate of a small heme was 1,400 s^−1^ at room temperature. The X-ray analyses revealed that the tertiary structure of globin containing the smallest heme was closely similar to that of native protein despite extensive destruction of the specific heme-globin interactions. The functional analyses of O_2_ binding showed that the loose heme-globin contacts do not significantly affect the oxygen binding. On the other hand, the rearrangement of tetrapyrrole array and the non-planar deformation in porphyrin ring significantly affect the functional properties of myoglobin. These results, taken together, indicate that the essential factors to regulate the myoglobin function are hidden under the molecular shape of prosthetic group rather than in the nonbonded heme-globin contacts.

## 1. Introduction

Iron porphyrin is the prosthetic group for an important class of proteins and enzymes. Many hemoproteins exhibit different functions such as reversible oxygen binding, electron transfer, and drug metabolism. The surrounding globin has been suggested to modulate the reactivity of heme and more specifically the reactivity of heme iron through the heme-globin interactions. During the past decades, many studies appeared to understand the relevance of globin to protein functions. Myoglobin (Mb), a simple oxygen binding protein, was the first protein whose tertiary structure was resolved by x-ray crystallography [1]. Owing to the stability of Mb, the heme is removable, and the apoMb is reconstituted with artificial cofactors [2,3,4,5,6]. Mb reconstitution with modified heme is a complementary approach to the globin modification by the protein engineering [7] to modify the function.

**Figure 1:**
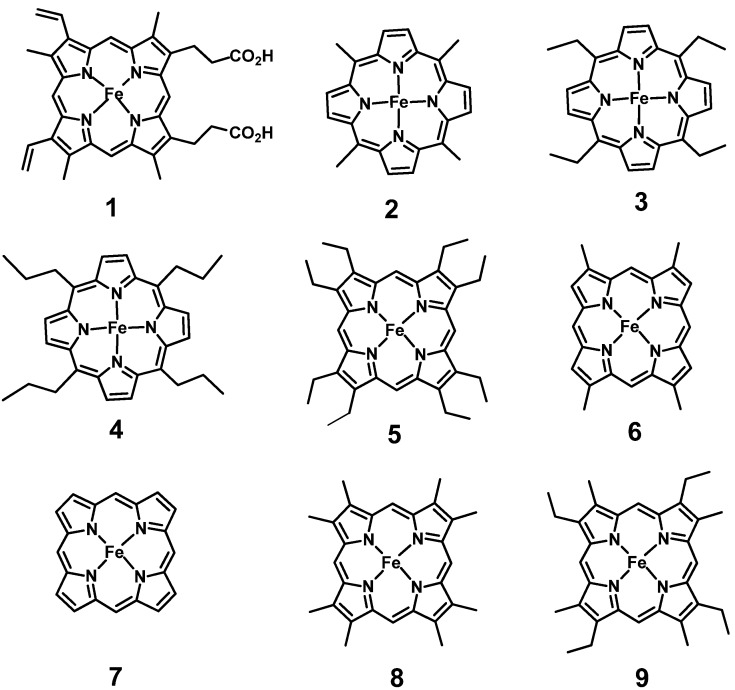
Structure of protoheme **1** and synthetic hemes **2**–**9**.

During the long history of structural analyses of hemoproteins, the heme in protein has never been appreciated as a mobile entity. This may arise from the static picture of heme as revealed by the X-ray crystallography for hemoproteins. La Mar and workers challenged the static view of the heme, and found in 1978 that protoheme (**1**, Figure 1) turned round on the α,γ-*meso* carbon axis in Mb [8,9]. Two orientations of the heme exist in the equal ratio immediately after the coupling with apoMb, and the main conformer dominates with time. Additional heme motion was found by our group in 1987 [10]. When the number of heme side-chains is reduced, the heme rapidly rotates about the iron-histidine bond in Mb. Since heme pocket is closely packed with globin residues, the heme has never been appreciated to be a mobile entity. This is a new type of example of protein fluctuation, and we have pursued the functional significance. We focus herein on the dynamic phenomenon of heme in Mb, and discuss the structural origin, thermodynamics, protein structure, and functional significance. 

Another topic in this review is the deformation of heme shape in Mb. Porphyrin is a cyclic tetrapyrrole with four pyrroles arrayed in a square manner. Recently, porphyrin isomers with a rectangular, trapezoidal, irregular quadrilateral were prepared, and their iron complexes have been used as the prosthetic groups of hemoproteins. We will examine how the shape-modified porphyrinoids control the O_2_ binding property of Mb.

## 2. Heme Rotation in Myoglobin

### 2.1. Myoglobin Reconstitution with Alkyl Hemin

Figure 1 shows the molecular structure of the prosthetic groups coupled with apoMb. The volume of the peripheral substituents in most of the hemes is smaller as compared with that in native protoheme **1**. Utilization of the synthetic hemes with various molecular shapes has twin advantages. First, the magnitude of the heme-globin contacts is controlled by selecting the number and molecular volume of the heme side chains. Secondly, the porphyrins are available in large amounts by organic synthesis.

In the conventional myoglobin reconstitution, the natural hemes with two propionate groups have been used [11]. This is because water soluble heme with propionate groups is essential to perform the coupling with apoMb in aqueous buffer solution. On the other hand, alkyl hemes **2**–**9**, without hydrophilic side chains and insoluble in water, have not been employed as the prosthetic groups of hemoprotein despite the potential utility. We employed water-miscible organic solvents to overcome the insolubility of alkyl heme in water. The typical procedure for Mb reconstitution with etioheme **9** is as follows [12]. Crude apoMb, prepared from 100 mg of native Mb by the acid-butanone procedure [11], was dialyzed overnight against cold 3 L of 10 mM *bis*-Tris buffer, pH 6.7 to remove 2-butanone. EtiohemeFe(III)Cl **9** (5 mg, 1.5-fold molar excess) was dissolved in hot dimethyl sulfoxide (3 mL; oil bath at 120 °C). The hemin solution was titrated to the string apoMb solution (ca. 30 mL) at pH 6.7 and 4 °C, and the mixture was dialyzed overnight against cold 3 L of 10 mM *bis*-Tris buffer at pH 6.7 to remove the organic solvent. The dialyzed solution was applied to a short column (1.5 cm × 5 cm) of carboxymethyl cellulose (Whatman, CM-52) equilibrated with 10 mM *bis*-Tris, pH 6.5. The purified ferric Mb was eluted with 0.1 M *bis*-Tris or Tris buffer at pH 7.0 in a cold room [12].

As the water-miscible organic solvents to dissolve alkyl hemins **2**–**9**, *N,N*-dimethylformamide, pyridine, methanol, ethanol, acetone, and their mixtures may be also used. It is important to solubilize hemin completely. A small volume of organic solvent is added to a weighed amount of hemin, the solution stirred well in a hot oil bath, and then centrifuged. These procedures are repeated until no precipitates are found at the bottom of centrifugation tube; complete dissolution of hemin in the organic solvents is essential for successful Mb reconstitution.

### 2.2. Detection of the Heme Rotation about the Iron-Histidine Bond

Prior to Mb reconstitution with alkyl hemins, we examined the incorporation of octaethylhemin **5** with eight ethyl groups on the heme periphery. The bulky alkyl heme was found to be introduced into the heme pocket to afford Mb**5** with normal oxygen binding property [13]. The observation suggested that apoMb would be capable of coupling with less bulky alkyl hemes such as **2**, **3**, and **4**. Another important implication is that the propionate groups in protohemin **1** are not always required for the Mb reconstitution. Encouraged with the successful preparation of Mb**5**, we attempted the coupling of a series of *meso*-tetraalkyl hemes **2**–**4** to modify systematically heme-globin contacts. We used proton NMR spectroscopy to monitor the modified hemes in Mb [10] because NMR serves as a powerful tool to analyze the paramagnetic center of hemoproteins [14].

**Figure 2:**
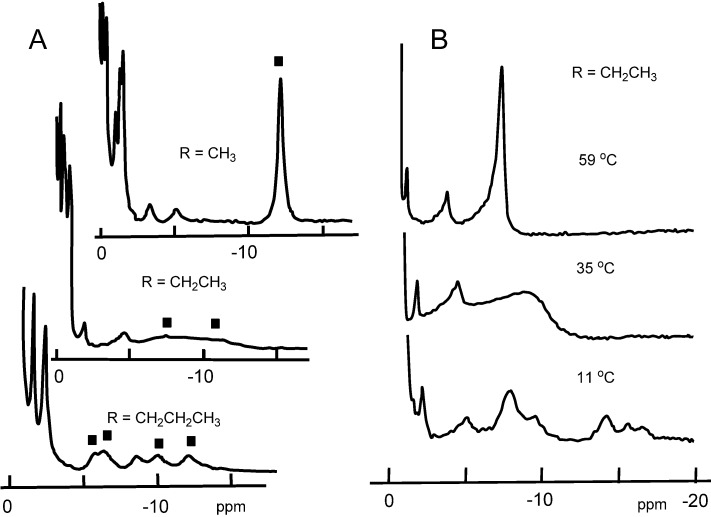
The 300 MHz proton NMR spectra of the reconstituted Mbs in ferric low-spin (*S* = 1/2) state. (**A**) Cyanomet complexes of the Mbs containing hemins **2**, **3**, and **4** with methyl, ethyl, and *n*-propyl side chains, respectively. The pyrrole protons are labeled with small squares. Temperature, 25 °C; (**B**) Temperature dependent NMR spectra of Mb**3**.

**Figure 3:**
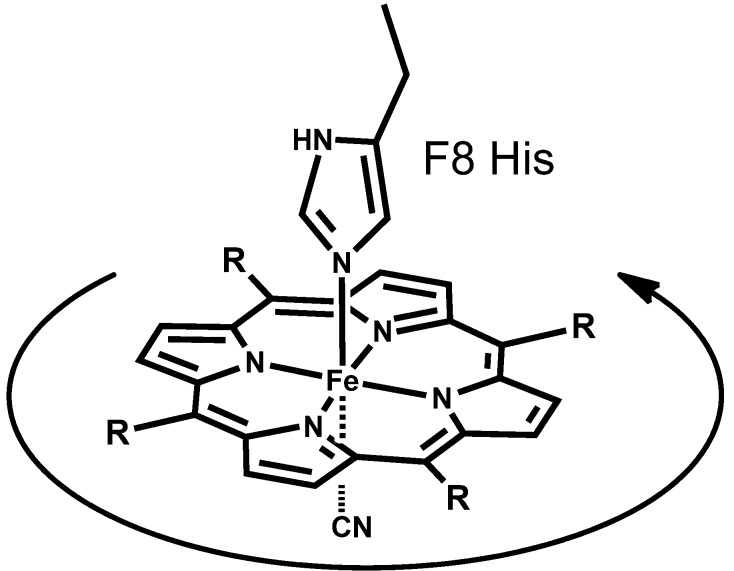
Heme rotation about the iron-histidine bond in Mb.

Figure 2A shows the paramagnetic proton NMR spectra at 25 °C of Mb**2**, Mb**3**, and Mb**4** coordinated with CN^-^ as the heme ligand [9]. The proton signals of the four pyrrole rings, labeled with squares, appeared in the upfield region owing to the paramagnetic iron [14]. One notable observation is that the eight pyrrole protons at heme periphery appeared as a single peak (Mb**2**), two broad peaks (Mb**3**), and four signals (Mb**4**) with elongation of the substituents. The systematic NMR transition may be correlated with the ligand exchange between H_2_O and CN^-^ at the sixth coordination site. However, the careful CN^−^ titration to Mb**4** indicated no NMR line broadening suggesting that the NMR transition in Figure 2A is not due to the ligand exchange, but comes rather from the dynamic process of the heme itself. This interpretation is supported from the temperature dependent NMR for Mb**3** bearing a heme of intermediate size. Figure 2B shows that the two broad signals of Mb**3**, as observed at 25 °C in Figure 2A, coalesced into a single peak at 59 °C, and split into four signals at 11 °C. The process was independent of the CN^-^ concentration. The spectra of Mb**3** at 59 °C and 11 °C were similar to those of Mb**2** and Mb**4** at room temperature, respectively. These observations demonstrated that the heme was dynamically rotating about the iron-histidine bond (Figure 3). The heme rotation as revealed by NMR for solution Mb is in sharp contrast with the results of X-ray crystallography that shows a static heme in protein crystal. This is a novel and typical example of the structural difference of protein in the solution and crystalline states. From the observation that the least bulky heme **2** is fairly mobile in Mb, we concluded that the primary role of heme substituent is prevention of heme rotation in the protein cavity. Heme rotation was supported by La Mar *et al.* from the NMR results of nuclear Overhauser effect [15].

### 2.3. Analysis of Heme Rotation Rate

**Figure 4:**
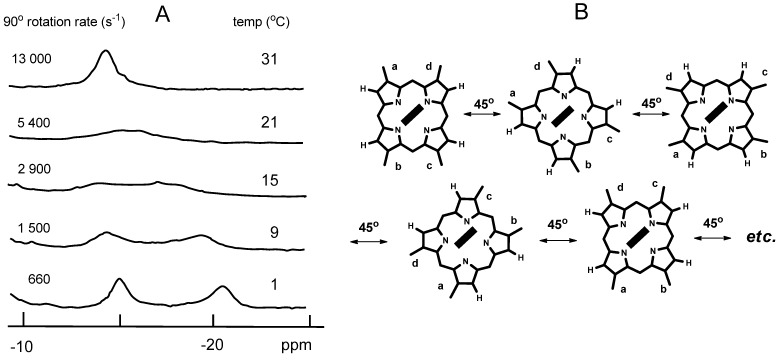
Proton NMR spectra of the CN^−^ complex of Mb**6** in ferric low-spin (*S* = 1/2) state at 300 MHz. (**A**) Pyrrole proton region. The chemical exchange rates are for the 90° rotation; (**B**) Pathway to interconvert the rotational isomers of hemin **6**. The filled rectangles denote the proximal histidine planes.

We reconstituted Mb with 1,4,5,8-tetramethylhemin **6** to estimate the rotation rate. Utilization of heme **6** is advantageous over hemes **2**, **3**, **4** because it has a lesser the number of pyrrole-protons than hemes **2**–**4** to make the NMR analysis easier. In addition, the four heme methyl groups in heme **6** are also used as the NMR probes. Figure 4A shows the temperature-dependent NMR spectra of Mb6 in the high-field region [16]. The peaks four pyrrole-protons of heme **6** appeared in the −15 to −20-ppm region. The signal pattern is markedly dependent on temperature; the two broad peaks at 15 °C coalesced into a single peak at 31 °C. The four methyl signals of **6**, appeared in the +20 to +30-ppm low field region, also exhibited the same NMR changes as the pyrrole proton signals in Figure 4A [16].

Figure 4B illustrates the rotational pathway of hemin **6** in the heme pocket. From the analysis of the interchange model of pyrrole-protons, the rotational rates shown in Figure 4A were obtained. The 90° rotation rate of 5,400 s^−1^ at 21 °C corresponds to 1,350 s^−1^ for 360° rotation. It is important to note in the scheme of Figure 4B that the direct 360° rotation is not involved, and that rotation occurs in a stepwise manner. This scheme is consistent with the clever NMR experiments reported by the group of Simonneaux [17,18]. They reconstituted Mb with small hemes of 3,7-diethyl-2,8-dimethylhemin and 3-ethyl-2-methylhemin. These hemes, substituted with only four or two peripheral sites by ethyl and methyl groups, did not rotate about the iron-histidine bond despite the small molecular size. Their results demonstrate that dynamic heme rotation occurs not through the direct 360° rotation but the stepwise heme hopping. From the analysis of the rotational rates with the Eyring plots for Mb6, the thermodynamic parameters of Δ*H* = 16.2 kcal mol^−1^ and Δ*S* = 13.7 cal mol^−1^K^−1^ were obtained [16]. The relatively large Δ*S* value was consistent with the large NMR spectral transition which occurred in narrow temperature ranges as illustrated in Figure 4A. The large Δ*S* further indicates that the heme rotation is induced by the fluctuation of numerous heme-globin contacts.

### 2.4. Crystallographic Structure of Mb with Rotating Heme

X-ray structural analysis of native Mb revealed that the heme was surrounded by many globin side chains [19]. In view of the proposed role of the heme-globin contacts in preserving the overall protein structure, it will be interesting to examine the entire Mb structure under loose heme-protein contacts. The small hemes in Figure 1 provide the unique opportunity to evaluate the role of the van der Waals contacts of heme in maintaining the tertiary structure. We prepared the Mb reconstituted with the simplest heme, iron porphine **7**. This heme, owing to the complete removal of the side chains, totally disrupts the heme-globin contacts when it is placed in protein cavity. How does the smallest heme **7** binds to apoMb, and how does the globin folds under extremely modified heme-protein contacts? We analyzed the NMR spectrum and the X-ray crystallographic structure to provide answers to these issues [20].

**Figure 5:**
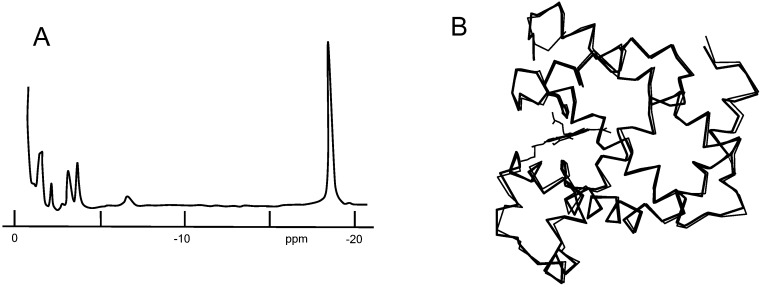
The MbCN^-^ reconstituted with heme **7** in ferric low-spin (*S* = 1/2) state. (**A**) Proton NMR of the pyrrole proton region at 300 MHz and 20 °C; (**B**) Superposition of the structure of Mb**7** (heavy lines) and native Mb**1** (thin lines).

Figure 5A shows the proton NMR spectrum of Mb**7**, where the eight pyrrole protons of heme **7** are observed as a large peak at −18.7 ppm. Appearance of the single peak indicates that the four pyrrole rings in heme **7** became equivalent due to rapid heme rotation about the iron-histidine bond. The line width of the pyrrole proton signal is about a half as that of Mb**2** (Figure 1), suggesting accelerated motion of heme **7** as compared with heme **2**. On the other hand, the heme was found to be fixed in the crystalline Mb**7** as shown in Figure 5B. The mobility difference between the solution and crystalline Mbs is attributable to the loss of globin flexibility in the latter. Another important observation in the crystalline Mb**7** was that the overall tertiary structure was least perturbed, as demonstrated by the superposition of the tertiary structures of Mb**7** and native Mb**1** in Figure 5B. The average C_α_ deviation between the two Mbs was only 0.52 Å. The fundamentally conserved molecular structure the most unexpected because severe globin deformation is reasonably inferable from the extremely loose fit of **7** in the heme pocket. We have obtained closely related results of conserved globin structure in the Mbs containing hemes **2**, **3**, and **4** [21,22], where systematic loosening of the heme-globin contacts did not much alter the globin fold.

Breslow *et al.* [23] demonstrated that the protoporphyrin without central iron can be complexed with apoMb to produce stable iron-free Mb. They pointed out the tertiary structural similarity between native Mb and iron-free protoporphyrin·Mb. Mb**7** is a unique counterpart of the iron-free Mb with regard to the presence of the iron-histidine bond and the absence of specific heme-peripheral contacts. These results for Mb**7** and iron-free Mb, taken together, indicate that neither the specific heme peripheral contacts nor the iron-histidine bond is essential to maintain the tertiary structure of Mb. Complex globin fold in Mb is more inherent in the amino acid sequence than is generally believed.

### 2.5. CO Binding to the Mb Reconstituted with Small Hemes

Since the heme is the ligand binding site of Mb, the functional consequences of heme rotation is a matter of importance. We analyzed the CO binding profiles of the Mbs containing natural heme **1** and small hemes **2**, **3**, **4** and **7** using flash photolysis and stopped-flow methods [24]. The kinetic and equilibrium parameters are compiled in Table 1.

**Table 1:** Kinetic constants of CO binding and vacancy space for native and reconstituted Mbs.

Mb	*k*_on_ (μM^−1^s^−1^)	*k*_off_ (s^−1^)	*K* (μM^−1^)	Vacancy space (Å^3^) ^a^
Mb**7**	3.6	0.583	6.2	399
Mb**2**	12.6	0.323	39.0	426
Mb**3**	15.1	0.356	42.0	438
Mb**4**	7.4	0.180	41.0	411
Mb**1**	0.7	0.016	44.0	385

^a^ Vacancy space, a measure for wideness of the distal heme pocket, was calculated from the X-ray results [20,21,22].

Table 1 indicates that the CO association rates (*k*_on_) increased by 5- to 20-fold, and that the dissociation rates (*k*_off_) raised by 10- to 36-fold upon replacing protoheme **1** with the small hemes. These features could be explained on the basis of the molecular structures of reconstituted Mbs. The side chain of Arg45 protruded from the heme vicinity to the solvent region, and the small hemes were tilted by the interactions of the *meso*-alkyl side chains of heme with surrounding peptide [20,21,22]. This resulted in formation of the larger vacancy space for CO movement (Table 1). These characteristic structures of the Mbs reconstituted with hemes **2**–**7** indicate that the CO ligand can more easily migrate in the heme cavity in comparison with native Mb1.

Despite the variation in the kinetic parameters, the equilibrium CO affinities of Mb**2**, Mb**3**, and Mb**4** are almost identical with native Mb. On the other hand, Mb**7** containing the tiny heme exhibits 7-fold reduced CO affinity. Analysis of the local heme translational motion, derived from anisotropic temperature factors, provided an account for this observation. The vibration amplitude of heme **7** (0.9 Å) was larger than 0.4 Å for hemes **2**–**4** [23]. Increased motion of heme **7** was consistent with instability of the coordinating CO or a larger dissociation rate shown in Table 1. Only in Mb7, the atomic distance between Val68 methyl groups and heme *meso*-carbon was as close as 4 Å. In view of a large mobilization amplitude (0.9 Å), the atomic distance between Val68 and heme **7** could become less than that of van der Waals interactions at several moments. This may cause the shift of Val68 methyl groups toward CO, the destabilization of Fe-CO bond, and an increase in the *k*_off_ rate for CO.

It is also notable that the variation in the *k*_on_ and *k*_off_ is not directly correlated with the side-chains volume of hemes **2**–**4**. Different kinetic parameters and equilibrium affinities primarily come from the larger vacancy space in Mb**2**–**4**, as compared with native Mb**1** (Table 1), rather than the heme rotation about the iron-histidine bond.

### 2.6. Function of the Mb Bearing Rotating Heme

**Figure 6:**
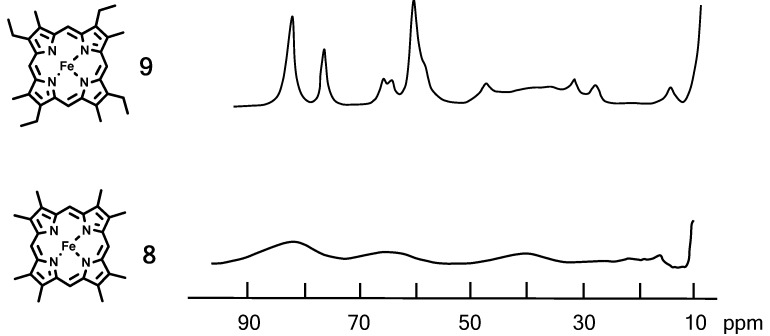
Proton NMR spectra of aquomet derivatives of Mb**8** and Mb**9** in ferric high-spin (*S* = 5/2) state at pD 7.0 and 25 °C. The four methyl signals in Mb**9** are at 82.8, 82.8, 76.7, and 59.9 ppm. Mb**8** shows no sharp heme methyl signals to reflect heme rotation.

Evaluation of heme rotation to the function of Mb is essential. Although the CO binding to the Mb**2**–**4** showed unique kinetic profiles, the results primarily reflected the vacancy space of the heme distal site. Influence of heme rotation was more elaborately examined with a pair of Mbs containing mobile heme **8** (octamethylheme) [24] and immobile heme **9** (etioheme) [12]. The substitution pattern of these hemes is identical with that in native protoheme **1**, and the molecular volumes of hemes **8** and **9** are similar to each other. The proton NMR spectra of Mb**8** and Mb**9** in ferric high spin (*S* = 5/2) state are compared in Figure 6. An interesting observation is that the four heme methyl peaks in Mb**9** were identified in the 60–80 ppm region while the eight methyl signals in Mb**8** are not observable owing to intensive line broadening. These NMR results suggest that heme **9** is essentially static on the NMR time scale while heme **8** is rapidly rotating. Tran and coworkers noted a possibility that the heme **9** rotates about the iron-histidine bond with a rate of 0.9–27 s^−1^ depending on the oxidation/spin state of the iron [25]. Since the rotation of heme **9** is slow enough on the NMR time scale, the static picture of heme **9** is a suitable approximation.

In Table 2 are summarized the kinetic and equilibrium parameters of the O_2_ binding to Mb**8**-**9** and native Mb**1**. The association and dissociation rates are almost identical between Mb**8** and Mb**9**, suggesting that the heme rotation does not directly affect the function. Resemblance between Mb**8** and Mb**1** further indicates that absence of heme propionate groups does not seriously affect the O_2_ binding kinetics. Close behavior of the two Mbs may be explained by elucidating the ligand binding process in Mb. Detailed kinetic analyses revealed that the O_2_ binding to Mb involves slow O_2_ diffusion into protein matrix before subsequent O_2_ migration to heme iron to form the iron-O_2_ bond [26,27]. Since the rate-limiting process occurs far from the prosthetic group, the O_2_ molecule does not perceive the rotating heme.

On the other hand, the heme rotation rate is in the order of 10^2^–10^3^ s^−1^ at room temperature [15] whereas the O_2_ association rate is ca. 10 s^−1^ (Table 2). Thus the heme motion could seriously affect the O_2_ dissociation process. Despite this expectation, the *k*_off_ rates for Mb**8** and Mb**9** are closely similar to each other (Table 2). These observations demonstrate that heme rotation alters the Mb function only slightly.

**Table 2:** Rate and equilibrium constants of the O_2_ binding to deoxy Mb.

Mb	*k*_on_ (μM^−1^s^−1^)	*k*_off_ (s^−1^)	*K* (μM^−1^)
Mb**8**	9.7	7.5	1.2
Mb**9**	8.4	8.5	1.0
Mb**1**	14	12	1.2

The results for Mb**8** with rotating heme have an intimate relevance to the fundamental aspects of heme-globin interactions in native Mb**1**. Protoheme **1** could be disordered in two orientations about the heme α,γ-*meso* carbon axis [8]. Livingstone *et al.* proposed that the heme **1** in a revered orientation enhances the O_2_ affinity by 10-fold due to the altered heme-globin contacts [28]. In contrast, Light *et al.* suggested the heme orientation disorder has little influence on the O_2_ binding [29]. Rotating heme **8** disrupts the specific heme-protein contacts much more than the heme orientation disorder. The results that the two Mbs with heme **8** (rotating) and heme **9** (static) have almost identical kinetic profiles (Table 2) support the view that the protoheme in normal and reversed orientations affects the Mb function only slightly [29].

Traylor [30] pointed out a distinct view of the heme orientation in Mb. He implied that the orientation of the proximal histidine plane relative to the iron-histidine bond may control the O_2_ binding. Smerdon *et al.* made a similar proposal from the investigation of Ser92 mutated Mb [31]. They remarked that the imidazole orientation against the diagonal nitrogen axis of heme could control the ligand binding kinetics through the breakage of the hydrogen bonding between the proximal histidine and Ser92 side-chain. In this case, the two overlapping effects of the hydrogen bonding and the imidazole orientation are difficult to separate. By contrast, the rotating heme **8** alters only the heme-histidine relative arrangement preserving the hydrogen bond. Mb**8** exhibits a simple kinetic trace of O_2_ binding which is strictly adjustable to monotonous exponential decay (Table 2). Rotating heme **8** is a statistic ensemble with several orientation conformers, similar to those depicted in Figure 4B. The apparently simple kinetic behavior of Mb**8** indicates that orientation of the proximal histidine against heme normal affects only moderately the O_2_ binding.

## 3. Core Modified Heme Analogues

**Figure 7:**
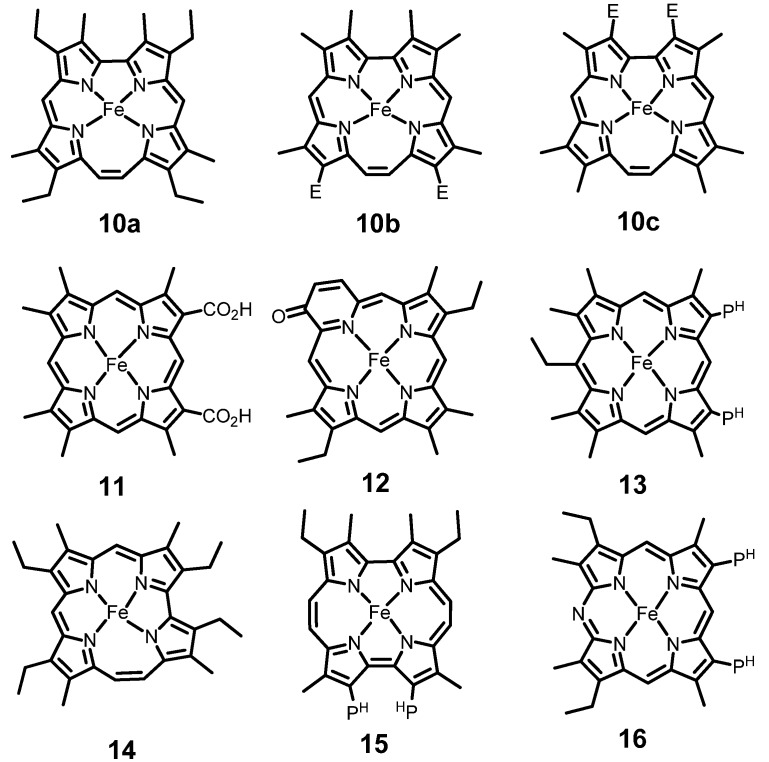
Iron porphyrin and the related compounds introduced into Mb pocket. *E* and P^H^ denote CO_2_C_2_H_5_ and CH_2_CH_2_CO_2_H, respectively.

We subsequently examined the influence by the heme molecular shape. Porphyrin is a cyclic tetrapyrrole connected with four *meso*-carbons. Regular porphyrin is thus designated as porphyrin-(1,1,1,1), where four numerals denote the number of *meso*-carbons. The notation indicates that the four pyrrole rings are made up in a square shape by four carbon atoms at the *meso*-bridges. Vogel and coworkers published in 1986 the first synthesis of a novel porphyrin isomer porphycene, a rectangular porphyrin-(2,0,2,0), and demonstrated that molecular shuffling of four pyrroles and four *meso*-carbons are possible [32]. Other porphyrin isomers with rearranged pyrrole rings and bridging carbons were prepared afterwards [33]. The deformed porphyrin isomers are useful tools to perturb directly the iron coordination site in porphyrin ring. Figure 7 shows the structure of iron complexes of isomeric porphyrins and related macrocycles which have been introduced into Mb.

Compound **10a** is an isomeric porphyrin-(2,1,0,1) called etiocorrphycene [34]. The coordination cavity in **10a** is distorted from square in ordinary porphyrin to trapezoid. The trapezoidal deformation critically affected the central iron to influence seriously the function of Mb. As shown in Table 3, the O_2_ affinity of Mb**10a** (8.2 × 10^4^ M^−1^) fell down to 1/12 as compared with the counterpart Mb**9** (1.0 × 10^6^ M^−1^) [11]. It is very likely that the deformed core in **10a** caused irregular iron-N(pyrrole) bonds which were not orthogonal to each other, thereby disturbing the movement of displaced iron toward the heme plane on O_2_ binding. The influence of the trapezoid deformation of heme is also evident in the example of Mb**10b**. Although both corrphycene **10b** [35] and regular heme **11** [36] commonly have two carboxyl groups, the oxygen affinity is significantly lower in the former (Table 3). This anomaly is also assigned to distortion in the equatorial coordination environment in **10b**, where the iron movement into heme plane on O_2_ ligation is disturbed. Another interesting observation is found in comparison of Mb**10b** with Mb**10c** [37]. Relocation of the two ester groups from the dipyrroethene part in **10b** to bipyrrole moiety of **10c** reduced down the O_2_ affinity of Mb to as low as 1/320 (Table 3). The phenomenon arises from the presence of the longer and shorter iron-N(pyrrole) bonds in the iron atom in the trapezoidal core of corrphycene. The two ester groups attached to the bipyrrole moiety affects to a larger extent the iron through the shorter iron-N(pyrrole) bonds to reduce the O_2_ affinity. Such an influence of the relocation of the ester groups, not observable for the iron in regular square hemes, is characteristic of trapezoidal corrphycene [37].

**Table 3:** Oxygen affinity of reconstituted Mbs with the porphyrinoids in Figure 7.

Mb	*K* (M^−1^)	Characteristic of coordination structure
Mb**10a**	8.2 × 10^4^	Corrphycene: Trapezoidal heme
Mb**10b**	4.5 × 10^4^	Corrphycene: Trapezoidal heme with two ester side chains
Mb**10c**	1.4 × 10^2^	Corrphycene: Trapezoidal heme with two ester side chains
Mb**11**	3.7 × 10^5^	Regular heme with two carboxyl groups
Mb**12**	0	Oxypyriporphyrin: Pyridine-substituted heme
Mb**13**	4.4 × 10^4^	Nonplanar heme
Mb**14**	1.3 × 10^7^	Hemiporphycene: Asymmetric heme
Mb**15**	1.1 × 10^9^	Porphycene: Rectangular heme with two propionate groups
Mb**16**	5.5 × 10^8^	Azaporphyrin: Regular heme with one *meso*-nitrogen

Additional influence of the macrocycle rearrangement is found in iron oxypyriporphyrin **12**, where one of pyrrole ring is replaced with a keto pyridine subunit [38]. In Mb**12**, the iron atom is surrounded by the four nitrogen atoms which are placed in irregular asymmetric quadrangle. Under the situation, the proximal histidine strongly hooks in the iron atom, and the in-plane shift of iron is essentially inhibited upon O_2_ binding. This results in a complete loss of O_2_ affinity of Mb**12**. Another mechanism to reduce the O_2_ affinity of Mb by heme core modification is found in heme **13** [39]. The *meso*-ethyl group in heme **13** deforms heme from planar to non-planar conformations, causing the iron atom to be displaced markedly from heme plane by the proximal histidine. This results in a stronger iron-histidine interaction. The largely displaced iron moves less smoothly upon the O_2_ binding, reducing the O_2_ affinity of Mb. The mechanism for Mb**13** [39] is analogous to that proposed for Mb**10a** and Mb**12**.

**Figure 8:**
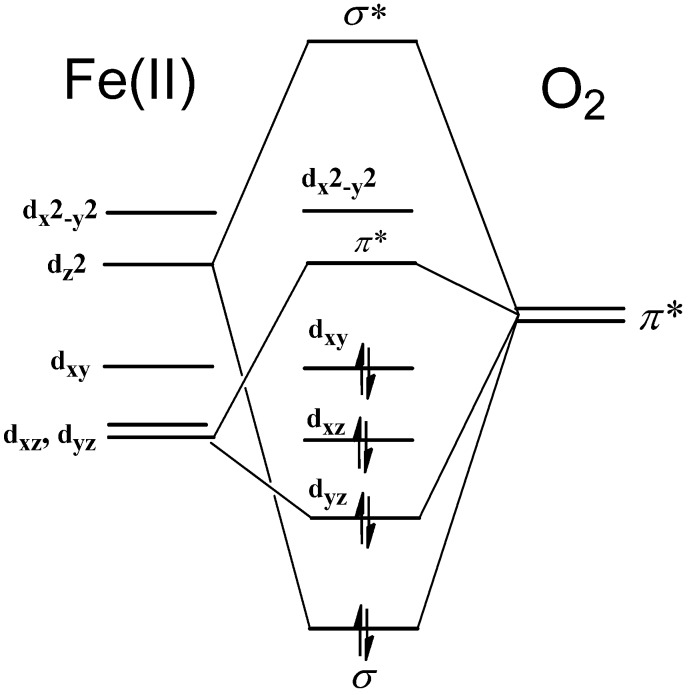
A qualitative molecular orbital scheme of the iron-oxygen complex.

Changed molecular shape of porphyrin not only reduce but also enhance the O_2_ affinity of Mb in some cases. The iron complex of hemiporphycene **14**, porphyrin-(2,1,1,0), is an asymmetric heme isomer. Mb**14** bearing iron hemiporphycene **14** [40] exhibits an O_2_ affinity of 1.3 × 10^7^ M^-1^; this is 13-fold larger than that of the reference compound Mb**9** [12] in Table 2. Relevant examples are found for the Mb reconstituted with the iron complexes of porphycene **15**, porphyrin-(2,0,2,0), and **16**, *mono*-azaporphyrin. The oxygen affinities of Mb**15** [41] and Mb**16** [42] are 1.1 × 10^9^ M^−1^ and 5.5 × 10^8^ M^−1^, respectively, which are much larger than 1.2 × 10^6^ M^−1^ of native Mb**1**. These results suggests participation of a structural factor other than the geometric strain in the N(pyrrole)-iron-N(pyrrole) bond angles. The ferric iron complexes of hemiporphycene **14** (7.882 Å^2^) and porphycene **15** (7.355 Å^2^) have narrower coordination cores than regular porphyrin (8.123 Å^2^) [43]. *mono*-Azaporphyrin **16** has also a contracted coordination core because nitrogen atom has a smaller atomic radius and narrower bond angle in comparison with carbon atom [42]. The contracted coordination holes of hemes **14**–**16** result in larger energy splitting between the d_z_^2^ and d_x_^2^_-y_^2^ orbitals of iron to stabilize the d_z_^2^ orbital, thereby strengthen the iron-O_2_σ-bond (Figure 8). Thus, the Mbs containing the iron porphyrinoids with narrower coordination cavities are expected to have enhanced O_2_ affinity. 

## 4. Conclusions

Mb is capable of accommodating variety types of synthetic hemes. The propionate groups present in natural porphyrins are not essential for the protein reconstitution. The alkyl porphyrins with diverse peripheral substituents and the cyclic tetrapyrroles with various molecular shapes are useful tools to perturb the heme-globin interactions in Mb. The NMR observation demonstrated that small hemes are fairly mobile about the iron-histidine bond. The X-ray crystallographic analyses, however, indicated that the tertiary structure of the reconstituted Mb was least perturbed by small hemes. In addition, the O_2_ reaction profile of the reconstituted Mb was almost identical with that in native Mb. On the other hand, the O_2_ binding in Mb was extremely sensitive to the rearranged tetrapyrrole array in porphyrin core. The O_2_ affinity increased by a factor of 10^3^ in corrphycene Mb**15**, and fell down to zero in oxypyriporphyrin Mb**12**. The overall O_2_ affinity changed by more than 10^7^-fold within native globin structure. The observations demonstrate that Mb reconstitution with modified hemes is a unique counterpart of protein engineering to regulate the Mb function. Accordingly, the artificial hemes should provide promising opportunity to design the heme-protein interactions and to create the new function of many other hemoproteins as well.
